# Effects of losartan and enalapril on serum uric acid and GFR in children with proteinuria

**DOI:** 10.1007/s00467-021-05045-4

**Published:** 2021-04-21

**Authors:** Charlotte E. Bryant, Azita Rajai, Nicholas J. A. Webb, Ronald J. Hogg

**Affiliations:** 1 Research and Innovation, Manchester University NHS Foundation Trust, Nowgen Building, Grafton St., Manchester, M13 9WU UK; 2 Centre for Biostatistics, Division of Population Health, University of Manchester, Manchester Academic Health Science Centre, Manchester, UK; 3 Kailua Kona, Hawaii USA

**Keywords:** Children, Chronic kidney disease (CKD), Hyperuricemia, Hypertension, Proteinuria

## Abstract

**Background:**

Studies have shown that losartan reduces serum uric acid in adults, unlike angiotensin-converting enzyme inhibitors. A previous study demonstrated that losartan and enalapril had comparable effects on proteinuria in children.

**Methods:**

We conducted a post hoc analysis of results from a prospective trial in which the proteinuria-reducing effects of losartan and enalapril were compared. We have now evaluated (a) the effects of these medications on SUA in 248 children with proteinuria and (b) the correlation between changes in SUA and eGFR.

**Results:**

SUA levels after 36 months were found to be increased when compared to baseline in both losartan and enalapril groups. The mean change in SUA from baseline was significantly different at 12 months between 23 hypertensive patients randomised to losartan (3.69% decrease [95% CI 11.31%, 3.93%]) and 24 randomised to enalapril (12.57% increase [95% CI 3.72%, 21.41%]), *p* = 0.007. This significant difference remained after 24, 30 and 36 months but was observed in the entire group of 248 patients only at 12 months. There was a statistically significant negative correlation between changes in SUA and changes in eGFR at each time point over 36 months.

**Conclusions:**

Losartan may have long-term beneficial effects on SUA and eGFR in children with proteinuria.

## Introduction

Hyperuricemia (HU) has long been associated with hypertension, diabetes, cardiovascular and kidney disease in adults [1, 2]. Increased serum uric acid (SUA) levels are common in patients with chronic kidney disease (CKD) due to declining glomerular filtration rate (GFR) and subsequent uric acid retention. However, in recent years, it has been reported that HU is also a contributing factor for both the development [3–7] and progression [8, 9] of CKD in adults, although some studies have found that this may not be true in patients with GFR values under 45 ml/min/1.73 m^2^ (i.e., CKD stages 3b and 4) [10, 11]. Although most of the studies have been conducted in adults, similar conclusions have also been reported recently in children [12–14]. The association of HU with accelerated progression of kidney insufficiency appears to be especially well documented in adult patients with IgA nephropathy [15–18].

Renewed interest in uric acid comes at a time when the rising prevalence of CKD necessitates the identification of early detection markers and strategies to delay or prevent progression to stage 5 CKD. The mechanisms responsible for progressive CKD are not fully understood; however, hypertension and proteinuria have been consistent candidates and pharmacologic interventions using ACEi and/or ARBs, with or without diuretics or low-sodium diets, have resulted in improved kidney outcomes in adults [19–24] and in children and adolescents with CKD [25–29]. Unfortunately, some of these treatment regimens are associated with electrolyte abnormalities, including HU. This has led to many investigators evaluating medications that reduce uric acid production [30–40] or increase excretion [40–49].

Of interest in this report is the uricosuric effect of losartan in children and adolescents who have CKD. Studies have demonstrated that whereas losartan lowers SUA in adults, this is not observed with other ARBs [43, 47] or ACE inhibitors, but there are no comparable paediatric studies. In order to address this issue, we performed a post hoc analysis of data obtained in a multicentre, prospective, randomised controlled trial (RCT) of children with persistent proteinuria in which it was shown that losartan and enalapril exhibited comparable long-term efficacy and tolerability in normotensive and hypertensive children with CKD of multiple aetiologies [28]. The post hoc analysis reported herein was conducted in 248 of the patients in this previous report. The goal was to determine if losartan and enalapril are associated with different effects on SUA levels in these patients, and to evaluate whether such changes in SUA correlate with changes in estimated GFR (eGFR).

## Methods

### Trial design

The initial study protocol that forms the background for this report (Merck Sharp & Dohme Corp. Losartan Protocol 326 NCT00568178) has been reported previously [27]. In the original trial, 306 children and adolescents < 18 years of age, with urine protein/creatinine ratios (UPCR) ≥ 0.3 (g/g), were enrolled in 50 participating centres. Normotensive (NT) patients were randomised to receive losartan (0.7–1.4 mg/kg/day) vs. placebo, whereas hypertensive (HT) patients received the same dose of losartan vs. amlodipine (0.1–0.2 mg/kg/day). The primary endpoint in this initial study was the change in UPCR from the time of study entry to the end of 12 weeks of therapy [27].

At the end of their involvement in this 12-week trial, the patients were enrolled in a subsequent long-term extension study which examined the effects of losartan vs. enalapril in 268 of the initial cohort of patients. Patients again were randomised 1:1, stratified by assigned treatment in the double-blind RCT. This extension study was prespecified to continue until 100 patients completed 3 years of follow-up. The primary endpoints were changes from baseline, defined in this post hoc trial as the values obtained at the end of the 12-week double-blind study, in both UPCR and eGFR during up to 3 years of treatment [28]. Full inclusion and exclusion criteria for the initial trial were reported previously [27]. The original study was conducted in accordance with the principles of Good Clinical Practice and approved by the relevant ethics review committees [12].

During the long-term extension study, investigators submitted data regarding patient blood pressure, ongoing medications, growth and adverse events that were measured at baseline, and every 6 months thereafter until the last study visit in the extension phase. Laboratory values including serum creatinine (SCr), serum uric (SUA) and serum cystatin C (CysC) levels (determined in a central laboratory) were also obtained at each visit.

### Eligibility criteria for this post hoc study

All subjects with uric acid measurements included in the Merck Sharp & Dohme Corp. Losartan Protocol 326 were eligible for inclusion in this post hoc analysis, with the exception of those receiving calcineurin inhibitors, such as cyclosporine A (CsA) and tacrolimus, since these medications are known to increase SUA levels.

### Outcome measures

The primary outcome measure was the change in SUA from baseline (last value in the double-blind 12-week RCT) to 6, 12, 18, 24, 30 and 36 months of treatment. The secondary outcome measure was the change in SUA in relation to kidney function as measured by a change in eGFR. Height measurements were obtained at each time point and eGFR was estimated using the formula detailed by Zappitelli and colleagues: (507.76 × e^0.003xheight^)/(CysC^0.635^ × SCr^0.547^ (μmol/L)) [50].

### Statistical analysis

The primary endpoint was the change in SUA on the natural logarithm scale from baseline (end of 12 weeks study) to each time point which was analysed using a mixed model repeated measure with time, time interaction by treatment (losartan/enalapril), stratum factor 1 (normotensive/hypertensive) and stratum factor 2 (prior treatment; losartan/amlodipine/placebo), gender and baseline log(SUA) as covariates and a random effect for the patient. The structure used for covariance matrix to represent correlation between outcomes at the different time points was banded 4 which assumes no correlation between outcomes with more than 4 time points apart. The treatment effects at each time point were estimated from this model along with associated 95% confidence intervals (CI). Additionally, treatment by blood pressure (BP group status (normotensive/hypertensive)) interaction terms was added to allow direct estimation of the treatment effects in the two subgroups and a Wald test of the relevant interaction term was used to ascertain whether the treatment had a differential effect according to baseline BP status. A significance level of 5% was considered and effect of multiple testing was in mind when interpreting the results. Analyses were performed in Stata C 13 (64-bit).

## Results

### Patient population

**Fig. 1:**
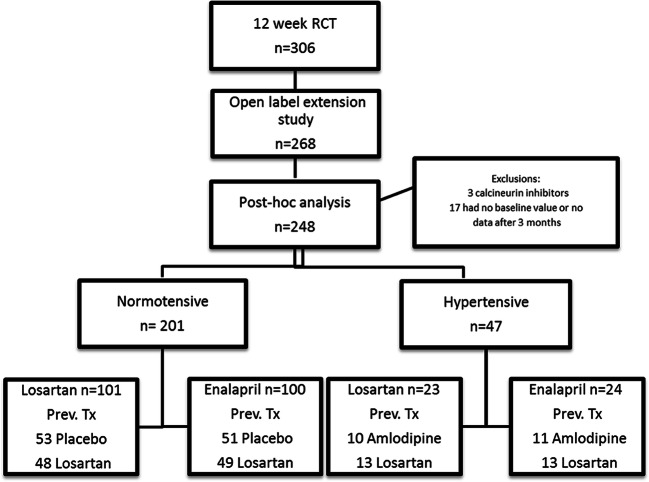
Study participant flow

Figure 1 summarises the study participant flow. A total of 306 participants were randomised in the initial 12-week RCT; 268 of these patients were re-randomised in the open-label extension phase, and data from 248 of these 268 patients were analysed in this post hoc study. Three of the 20 patients who were excluded were receiving calcineurin inhibitors and 17 had missing SUA values either at baseline or all 6–36 months’ time points. Missing SUA values were not related to treatment, previous treatment in the initial RCT or any particular participant characteristic at any time point.

Table 1 displays the baseline characteristics of the 124 patients receiving losartan (mean dose 1.17 mg/kg/day) and the 124 receiving enalapril (mean dose 0.26 mg/kg/day). Male participants outnumbered females but otherwise, there were no significant differences between the two treatment groups. Baseline SUA levels correlated positively with age (*p* < 0.001) and negatively with baseline eGFR values (*p* < 0.001) but were not significantly correlated with gender.

**Table 1:** Comparison of patient characteristics at baseline^1^ in the losartan and enalapril groups

Characteristic	Losartan *n* = 124	Enalapril *n* = 124	*p*-value
Gender, *n* (%)			0.04^+^
Female	44 (35.5%)	61 (49.2%)	
Male	80 (64.5%)	63 (50.8%)	
Age, median (interquartile)	10 (6.14)	10 (6.14)	0.70^
Race, *n* (%)			0.19^+^
Asian	20 (16.13%)	17 (13.71%)	
Black	6 (4.84%)	2 (1.61%)	
Multiracial	25 (20.16%)	40 (32.26%)	
White	68 (54.84%)	61 (49.19%)	
Other	5 (4.03%)	4 (3.23%)	
Blood pressure group, *n* (%)			0.99^+^
Normotensive	101 (81.5%)	100 (80.6%)	
Hypertensive	23 (18.5%)	24 (19.4%)	
Prior ACEI/ARB use, *n* (%)			0.44^+^
No	59 (47.58%)	52 (41.94%)	
Yes	65 (52.42%)	72 (58.06%)	
Previous treatment in RCT, *n* (%)			0.98^+^
Amlodipine	10 (8.06%)	11 (8.9%)	
Losartan	61 (49.2%)	62 (50.0%)	
Placebo	53 (42.7%)	51 (41.1%)	
eGFR at baseline^1^, mean (SD) ml/min/1.73m^2^	84.1 (37.4)	89.6 (40.1)	0.13*
SUA at baseline^1^, mean (SD) mg/dl	5.38 (1.77)	5.37 (1.87)	0.98*
Etiologies of proteinuria, *n* (%)			0.7^+^
Glomerular	60 (48.39 %)	57 (45.97 %)	
Reflux nephropathy	11 (8.87 %)	12 (9.68 %)	
Hemolytic uremic syndrome	11 (8.87 %)	17 (13.71 %)	
Alport syndrome	15 (12.1 %)	14 (11.29 %)	
Hypoplasia/dysplasia/aplasia	6 (4.84 %)	7 (5.65 %)	
Obstruction	3 (2.42 %)	1 (0.81 %)	
Other	6 (4.84 %)	2 (1.61 %)	
Unknown	12 (9.68 %)	14 (11.29 %)	

Abbreviations: *ACEI*, angiotensin-converting enzyme inhibitor; *ARB*, angiotensin II type I receptor blocker; *eGFR*, estimated glomerular filtration rate

^+^Chi-squared test

^Mann Whitney *U*-test

*t-test

^1^baseline = results obtained at the end of the 12-week RCT

### Changes in SUA levels in response to losartan or enalapril

#### Treatment effects when all patients were combined

When data from the 124 NT and 124 HT patients were combined, SUA levels after 36 months were found to be increased when compared to baseline in both losartan and enalapril-treated participants, with the losartan patients showing a smaller increase in SUA compared to the enalapril group. However, there was no significant difference observed in change in SUA levels between the losartan and enalapril groups except at the 12-month time point (*p* = 0.018). In an additional analysis, adding glomerular status did not alter the results substantially.

#### Different treatment effects observed in HT vs. NT patients

**Fig. 2:**
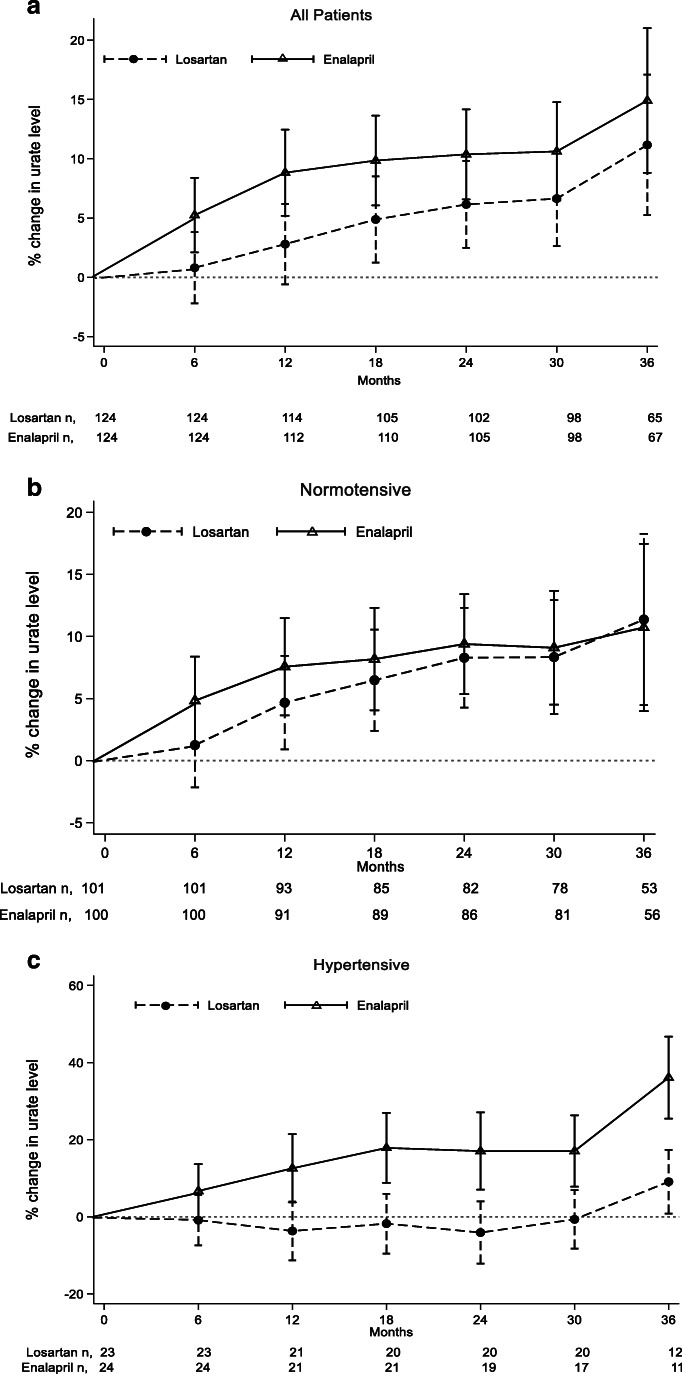
Percentage change in SUA over 36-month follow-up obtained after antilog transformation of the estimations from mixed-effect model with time interaction by treatment (losartan/enalapril), gender, stratum factor 1 (normotensive/hypertensive), stratum factor 2 (treatment at the first stage of study; losartan/amlodipine/placebo) and time as covariate, in **a** all patients, **b** normotensive patients and **c** hypertensive patients

Despite there being no difference in SUA levels between the two treatment groups, there were significant interactions between treatment and BP groups at each time point from 12 months onwards (*p*-values from Wald test: 0.48, 0.025, 0.015, 0.009, 0.021, 0.033). We therefore examined the SUA response to losartan and enalapril in the two BP groups separately. The mean change in log SUA was significantly different between the losartan and enalapril groups in HT participants at 12 months. Losartan group: −3.69% decrease (95% CI −11.31%, 3.93%) vs. enalapril group: 12.57% increase (95% CI 3.72%, 21.41%), *p* = 0.007(Table 2). This significant difference remained after 24, 30 and 36 months of treatment. This effect was not observed in NT participants. Figure 2 shows the estimated marginal mean percentage change in SUA from the mixed effect model in (a) all patients, (b) NT patients and (c) HT patients.

**Table 2:** Treatment effect of losartan and enalapril from mixed model repeated measure in hypertensive patients

Time	Losartan,* percentage change (95% CI)	Enalapril, * percentage change (95% CI)	Treatment effect, ^ 95% CI, *p*-value
6 months	−0.87 (−7.40, 5.66) *n* = 23	6.68 (0.31, 13.68) *n* = 24	0.073 (−0.0203, 0.1673) *p* = 0.125
12 months	−3.69 (−11.31, 3.93) *n* = 21	12.57 (3.72, 21.41) *n* = 21	0.16 (0.0429, 0.269) *p* = 0.007
18 months	−1.79 (−9.54, 5.96) *n* = 20	17.87 (8.78, 26.94) *n* = 21	0.18 (0.0705, 0.29) *p* = 0.001
24 months	−4.09 (−12.16, 3.97) *n* = 20	17.04 (7.01, 27.07) *n* = 19	0.19 (0.0773, 0.3210) *p* = 0.001
30 months	−0.67 (−8.25, 6.90) *n* = 20	17.04 (7.79, 26.0) *n* = 17	0.16 (0.0525, 0.275) *p* = 0.004
36 months	9.08 (0.83, 17.34) *n* = 12	36.12 (25.48, 46.75) *n* = 11	0.22 (0.1116, 0.3311) *p* = 0.0001

*Marginal mean percentage change (obtained after antilog transformation of the estimations) in SUA level in each treatment group in hypertensive patients

^Treatment effect from mixed effect repeated measure model (exact coefficients)

### Serum uric acid and estimated glomerular filtration rate

**Fig. 3:**
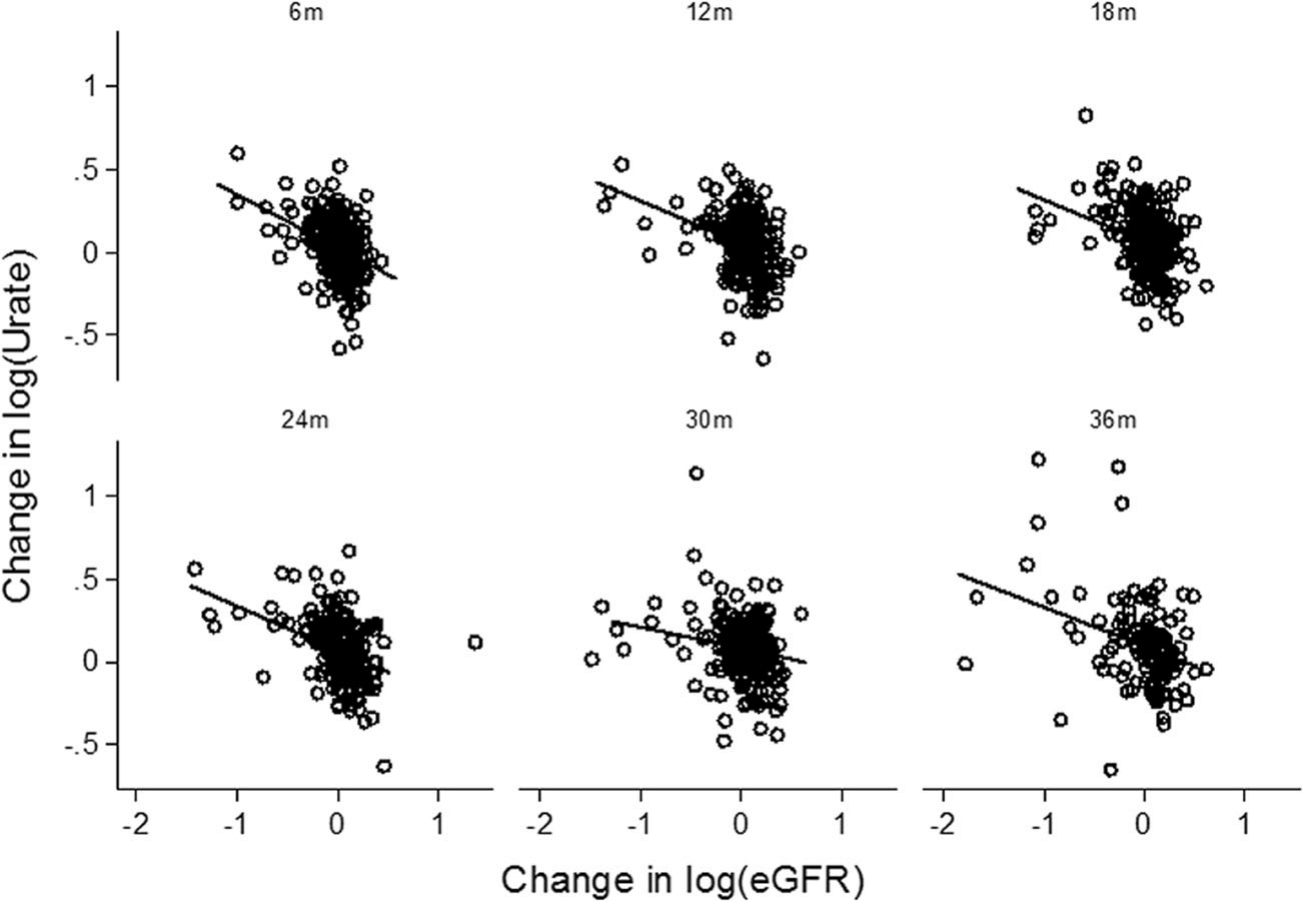
Change in log(SUA) vs. change in log(eGFR)

A mixed model repeated measure analysis with change in log(SUA) as dependent variable and time and time interaction by change in log(eGFR) as covariates showed a statistically significant negative correlation between changes in eGFR and changes in SUA at each time point over 36 months (all *p* < 0.001) (Fig. 3 and Table 3).

**Table 3:** Coefficients of log(eGFR) and their 95% CI from mixed effect repeated measure model of change in log(SUA) against change in log(eGFR)

Time	Coefficient of change in log(eGFR) (95% CI) *p*-value
6 months	−0.36 (−0.45, −0.26) < 0.001
12 months	−0.28 (−0.36, −0.21) < 0.001
18 months	−0.32 (−0.39, −0.24) < 0.001
24 months	−0.26 (−0.33, −0.19) < 0.001
30 months	−0.18 (−0.25, −0.11) < 0.001
36 months	−0.25 (−0.34, −0.17) < 0.001

## Discussion

This post hoc analysis of an investigator-led multicentre, prospective trial has demonstrated that children and adolescents with proteinuria and HT have significantly greater increases in SUA levels after receiving enalapril (*n* = 24; mean dose 0.26 mg/kg/day) when compared to patients receiving losartan (*n* = 23; mean dose 1.17 mg/kg/day) for up to 36 months. Furthermore, the change in SUA levels was correlated with change in eGFR. Unfortunately, it is not possible to conclude that the changes in SUA levels caused the changes in GFR since it is also known that changes in GFR can result in altered excretion rates of uric acid. The study did not demonstrate a significant relationship between treatment modality and SUA levels in NT patients with proteinuria.

The role of HU as a risk factor for CKD progression remains controversial, particularly since it is ubiquitous in CKD patients. However, studies from many countries have now shown that elevated SUA levels predict the development of CKD in subjects with normal kidney function [3–7]. Some reports have also demonstrated that higher SUA levels predict more rapid progression in patients with established CKD [6], but these findings have been contradicted by other studies [10]. One possibility for this apparent discrepancy is the variable CKD stages that are present in the patient groups that have been studied. For example, in the study reported by Srivastava et al., higher levels of SUA were independently associated with risk of kidney failure in patients with eGFR > 45 ml/min/1.73 m^2^ but not in those with eGFR < 30 ml/min/1.73 m^2^ [11]. Patients with eGFR 30–44 ml/min/1.73 m^2^ had an intermediate risk that did not reach statistical significance.

Evaluating whether HU is a biomarker or risk factor for CKD has been investigated using therapies that directly decrease the production of SUA, such as allopurinol and febuxostat. Although the SUA-lowering effect conferred by allopurinol slowed kidney disease progression in some HU subjects with CKD [18, 19], its effectiveness in slowing progression in patients with significant kidney impairment (CKD stages 3–4) has not been upheld in a recent placebo-controlled prospective clinical trial reported by Badve et al for the CKD-FIX Study Group [39]. The frequency of serious side effects was similar in the 2 groups (allopurinol 46%, controls 44%). A similar situation exists with febuxostat which appears to have a more profound effect on SUA levels than allopurinol [36–38]. Most small trials using febuxostat have shown promising results [36], but this was not the case with a recent large controlled trial reported by Kimura et al for the FEATHER Study Investigators [34]. It is interesting to note, however, that patients in this trial with CKD stage 3a (eGFR was approximately 52 ml/min/1.73 m^2^) appeared to do better than patients with CKD stages 3b or 4 (approximate eGFR 37 ml/min/1.73 m^2^). It remains to be seen whether there is a “point of no return” when looking at the impact of uric acid-lowering drugs on eGFR. The patients in our study had well-preserved GFR levels and hence would fall within the “good responder” category if the hypothesis described above is correct.

Mechanisms responsible for the purported nephrotoxic effects of uric acid have been examined in recent years. Some studies have shown that increased SUA levels result in increased RAS activity, kidney inflammation and impairment of renal autoregulation ultimately leading to glomerular hypertension and thus contributing to the initiation and development of kidney disease [51, 52]. Our data did not show an effect of glomerular status on change in SUA between the two groups. Experimental models have provided evidence for the mechanisms behind uric acid-induced damage. Raising uric acid levels in animal models and cell culture systems resulted in an increase in oxidative stress and endothelial dysfunction leading to systemic and glomerular hypertension and contributing to the progression of kidney disease.

The hypouricemic action of losartan is attributed to an inhibition of the human urate transporter 1 (URAT1) and resulting decline in urate reabsorption by the proximal tubule, the primary site of uric acid secretion and reabsorption. A recent study by Sun et al has shown how uricosuric responses may vary among patients as a result of *URAT1* gene polymorphisms [53].

The 3-year longitudinal follow-up of children and adolescents with a wide range of kidney disorders is a strength of our study, but a potential limitation is the lack of body mass index *z*-score data and comparatively small number of hypertensive patients (*n* = 47). The post hoc analysis of RCT data was not designed to investigate the effects of changes in SUA and kidney outcomes, and that is a further limitation. Nonetheless, the smaller increase in SUA levels seen with losartan in HT children and adolescents when compared to the greater increase in SUA associated with enalapril provides new and important data to support the use of losartan in children with CKD.

A further limitation is the lack of information regarding patients’ pubertal status over the study duration. The median age at enrolment was 10 years and despite no difference in baseline SUA between the two groups, it is plausible that the difference in gender ratio between the losartan (44/80) and enalapril (61/63) groups may have affected the final SUA after 36 months.

Our study did not evaluate whether the increase in SUA levels seen with enalapril would also be found with other ARBs or ACE inhibitors. It should also be noted that the comparable reduction in proteinuria we have found in short-term studies comparing lisinopril with losartan, or in long-term studies comparing enalapril with losartan, would not necessarily be duplicated with other ACE inhibitors, such as ramipril, the ACE inhibitor used so successfully by the ESCAPE Study Group [26, 29]. However, our findings suggest that RCTs comparing the effects of losartan and ACE inhibitors on SUA levels and kidney progression in children and adolescents with CKD should be considered further. This may be of special importance in situations where these medications are prescribed in association with diuretics or lowsodium diets. It will also be relevant to include a significant number of children without proteinuria in future trials since it is important to determine if losartan’s beneficial impact on SUA levels and GFR are independent of the effects on proteinuria. In addition, the inclusion of children with varying stages of CKD will be important in order to determine if the baseline level of GFR is an important determinant of the potential benefit of losartan on preservation, or even improvement in GFR levels.

## Declarations

### Disclaimer

Merck Sharp & Dohme Corp., a subsidiary of Merck & Co., Inc., Kenilworth, NJ, USA, did not provide any sponsorship for the project and had no role in the analysis of the data or the preparation of the manuscript.
